# Supplementary figures and images for: How representative are student convenience samples? A study of literacy and numeracy skills in 32 countries
Source: PLoS One. 2022 Jul 8;17(7):e0271191. doi: 10.1371/journal.pone.0271191 (PMC9269910; doi:10.1371/journal.pone.0271191)

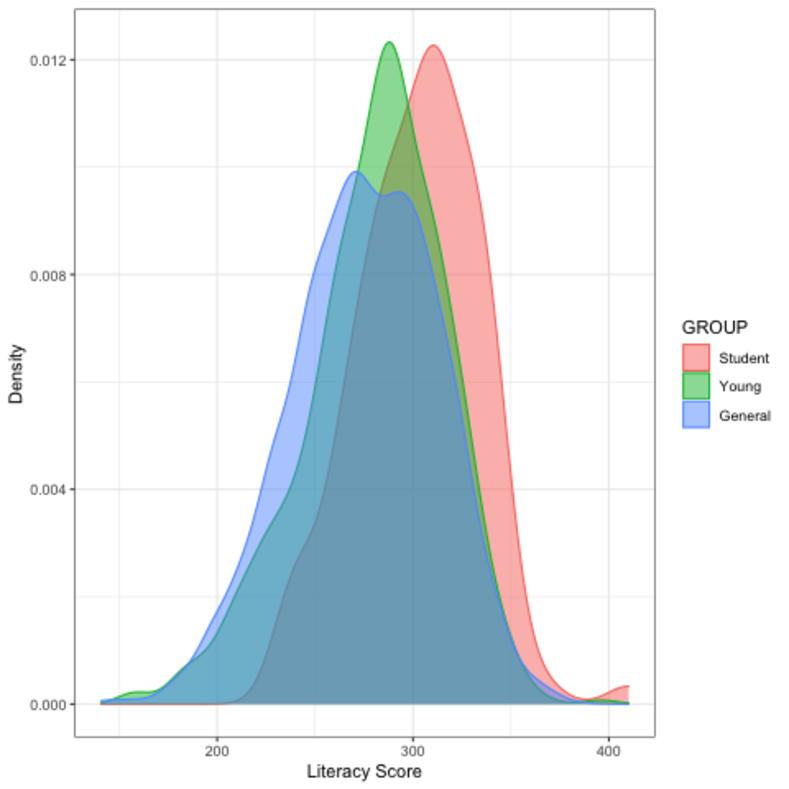

Supplement: S1 Fig — The red curve represents the Student sample, green represents the Young Sample, and blue represents the General sample. (TIF) [file pone.0271191.s001.tif]

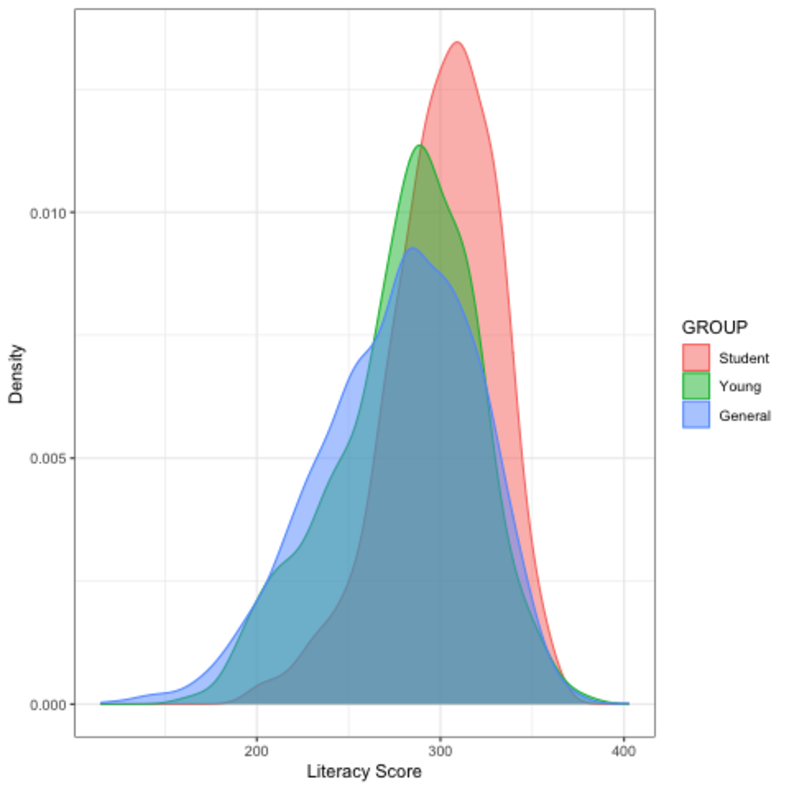

Supplement: S2 Fig — The red curve represents the Student sample, green represents the Young Sample, and blue represents the General sample. (TIF) [file pone.0271191.s002.tif]

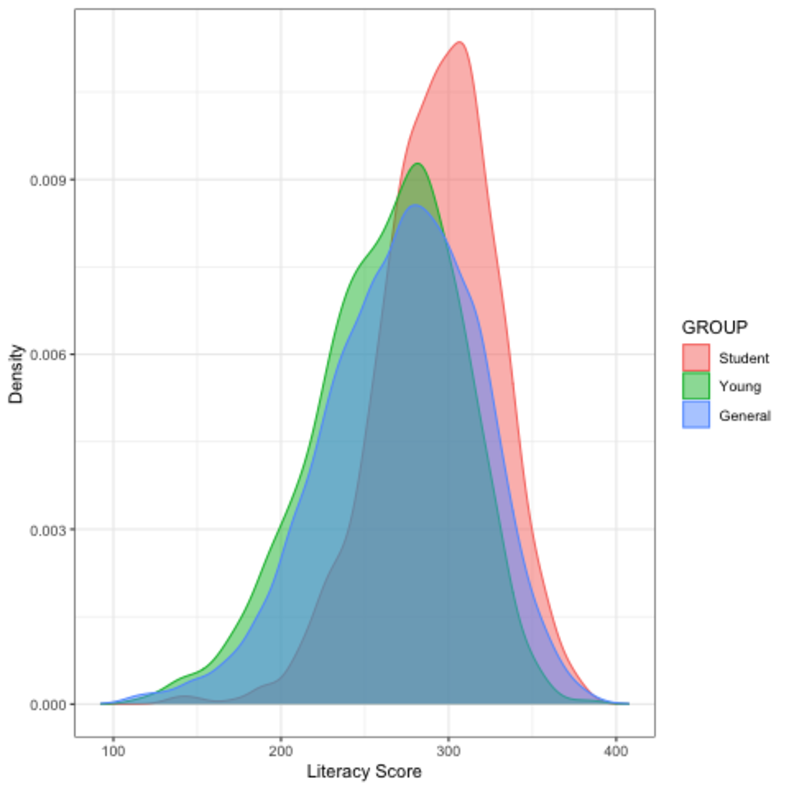

Supplement: S3 Fig — The red curve represents the Student sample, green represents the Young Sample, and blue represents the General sample. (TIF) [file pone.0271191.s003.tif]

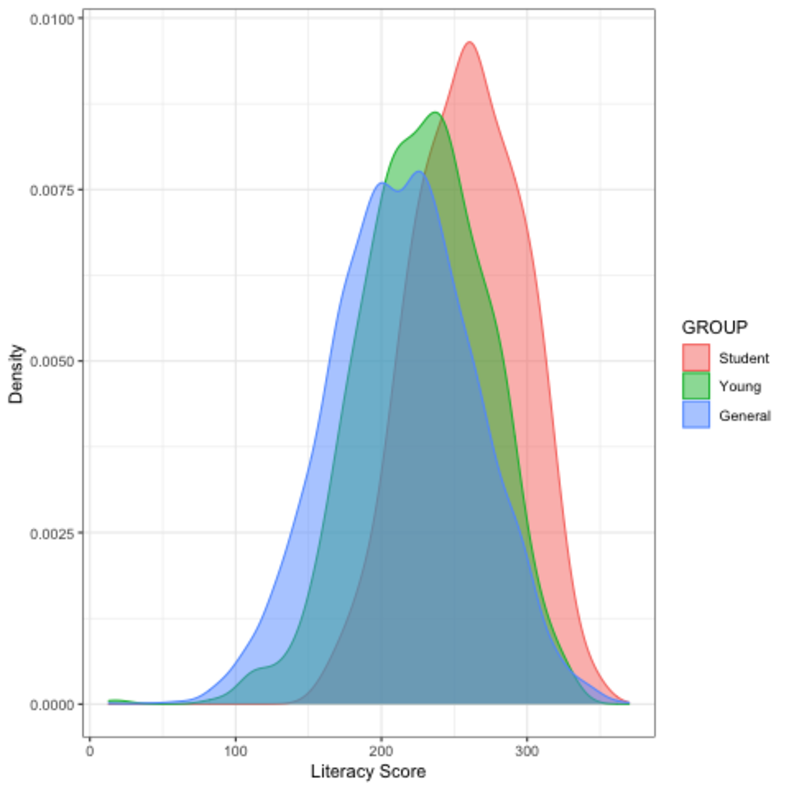

Supplement: S4 Fig — The red curve represents the Student sample, green represents the Young Sample, and blue represents the General sample. (TIF) [file pone.0271191.s004.tif]

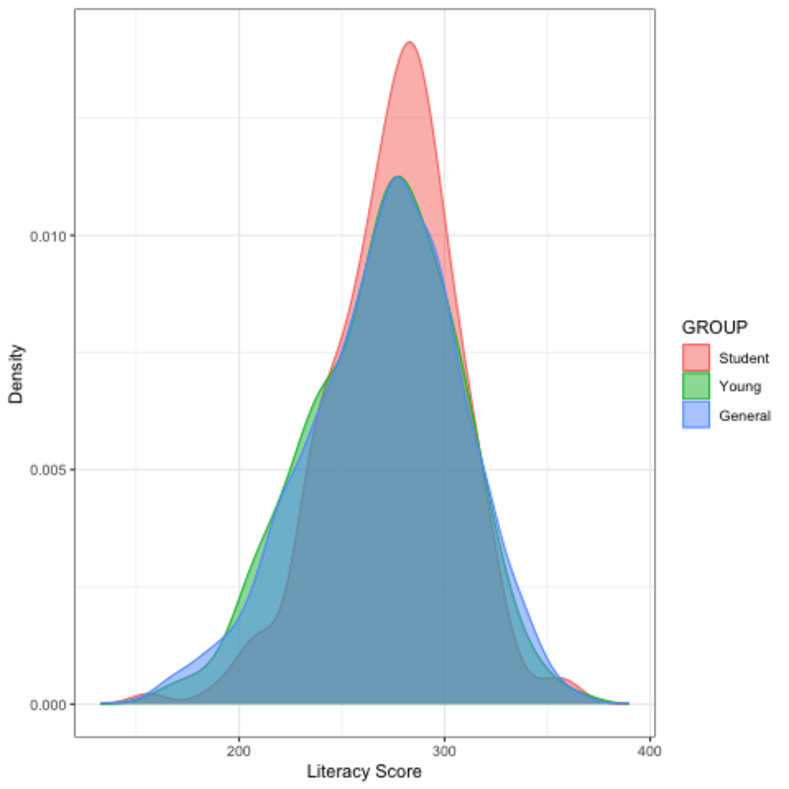

Supplement: S5 Fig — The red curve represents the Student sample, green represents the Young Sample, and blue represents the General sample. (TIF) [file pone.0271191.s005.tif]

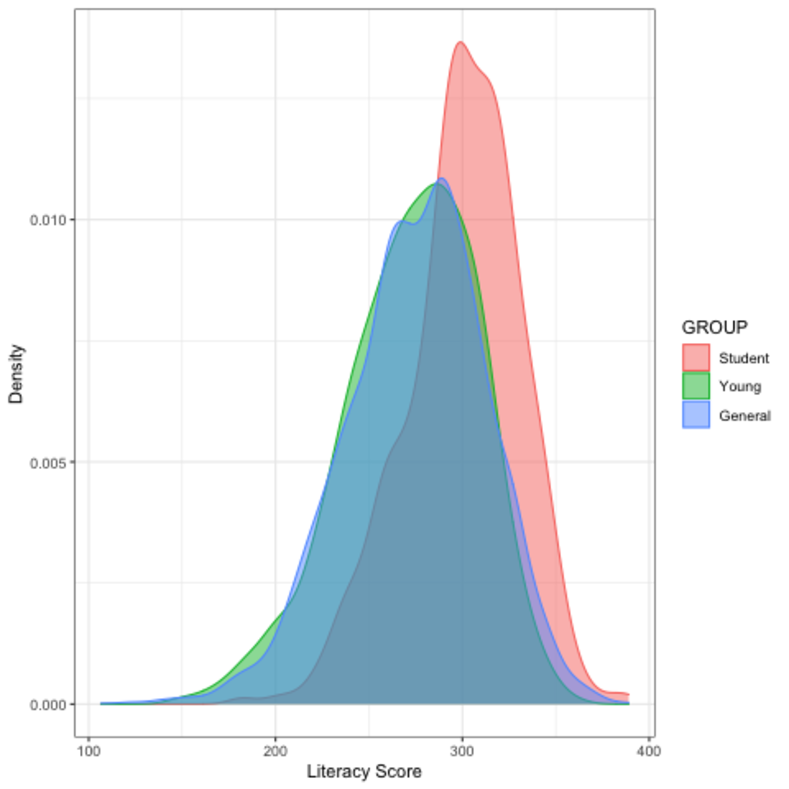

Supplement: S6 Fig — The red curve represents the Student sample, green represents the Young Sample, and blue represents the General sample. (TIF) [file pone.0271191.s006.tif]

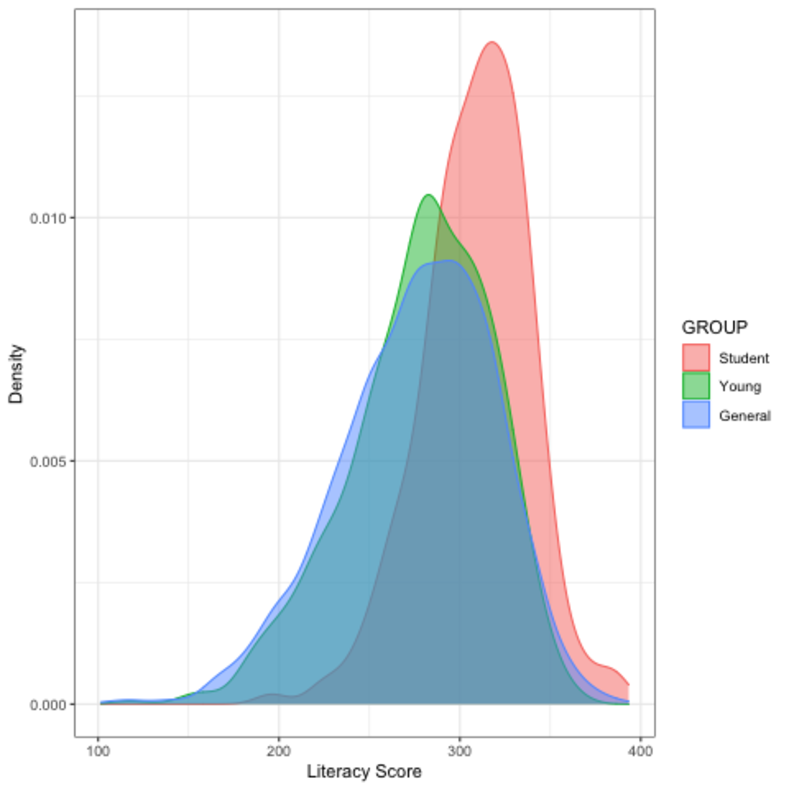

Supplement: S7 Fig — The red curve represents the Student sample, green represents the Young Sample, and blue represents the General sample. (TIF) [file pone.0271191.s007.tif]

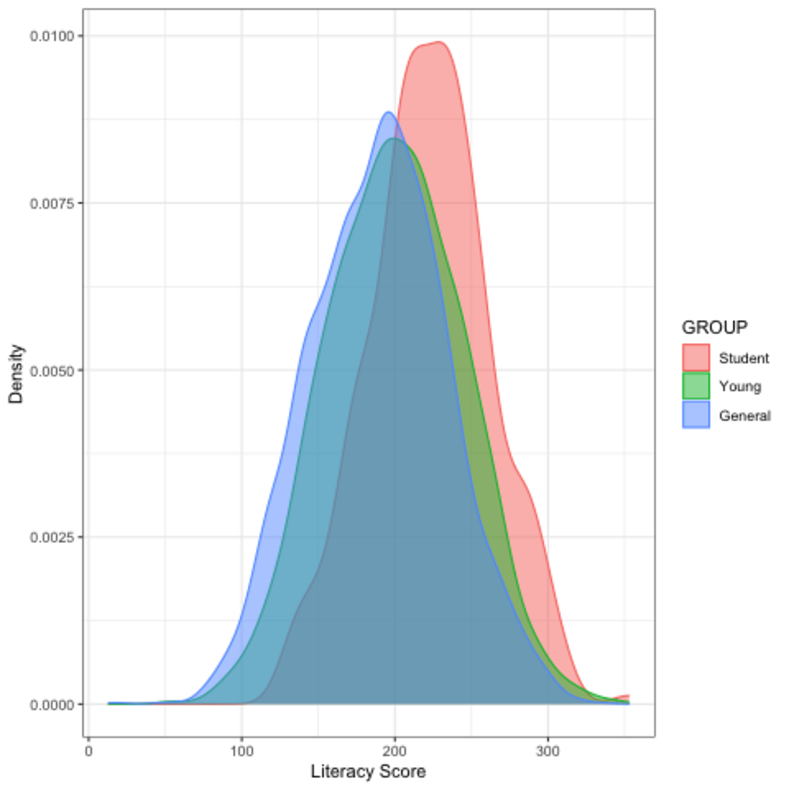

Supplement: S8 Fig — The red curve represents the Student sample, green represents the Young Sample, and blue represents the General sample. (TIF) [file pone.0271191.s008.tif]

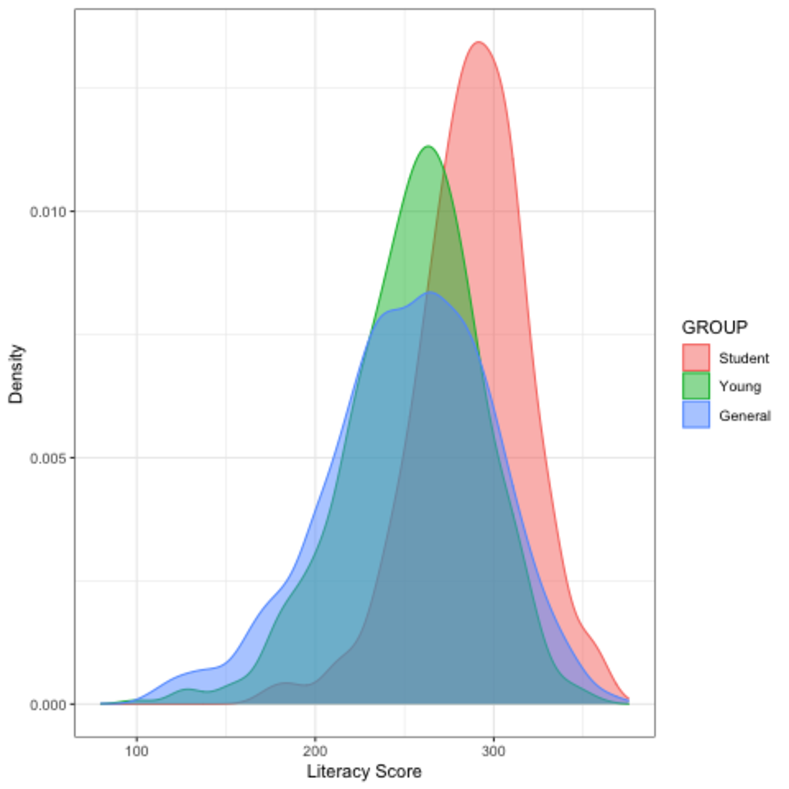

Supplement: S9 Fig — The red curve represents the Student sample, green represents the Young Sample, and blue represents the General sample. (TIF) [file pone.0271191.s009.tif]

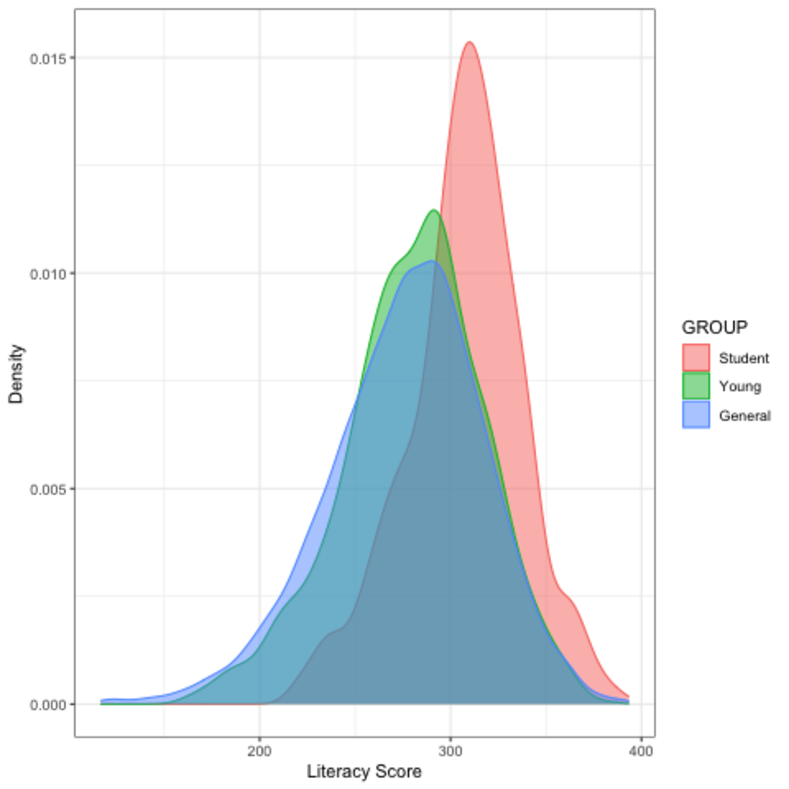

Supplement: S10 Fig — The red curve represents the Student sample, green represents the Young Sample, and blue represents the General sample. (TIF) [file pone.0271191.s010.tif]

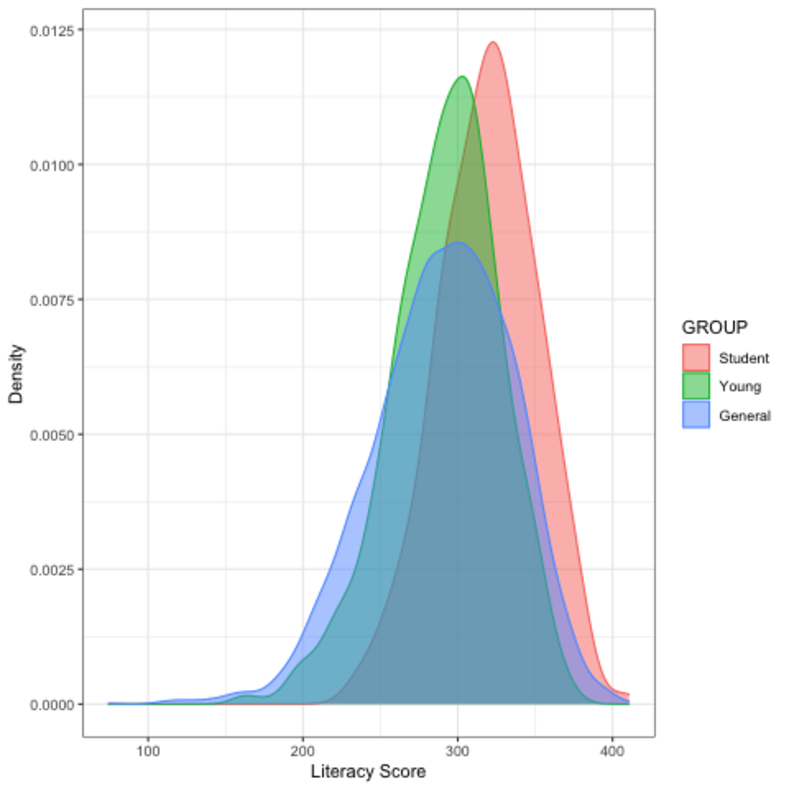

Supplement: S11 Fig — The red curve represents the Student sample, green represents the Young Sample, and blue represents the General sample. (TIF) [file pone.0271191.s011.tif]

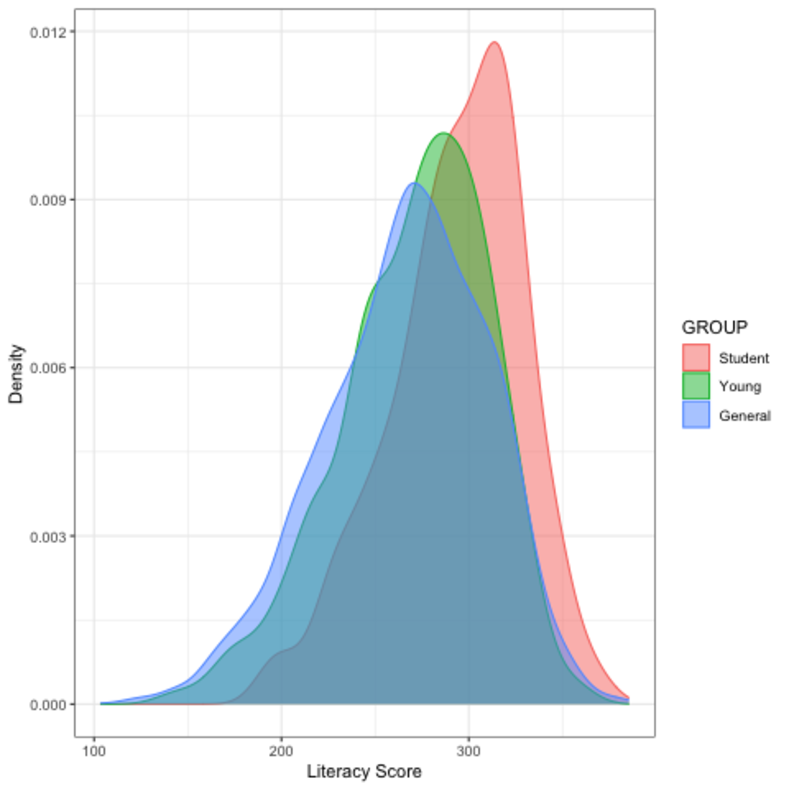

Supplement: S12 Fig — The red curve represents the Student sample, green represents the Young Sample, and blue represents the General sample. (TIF) [file pone.0271191.s012.tif]

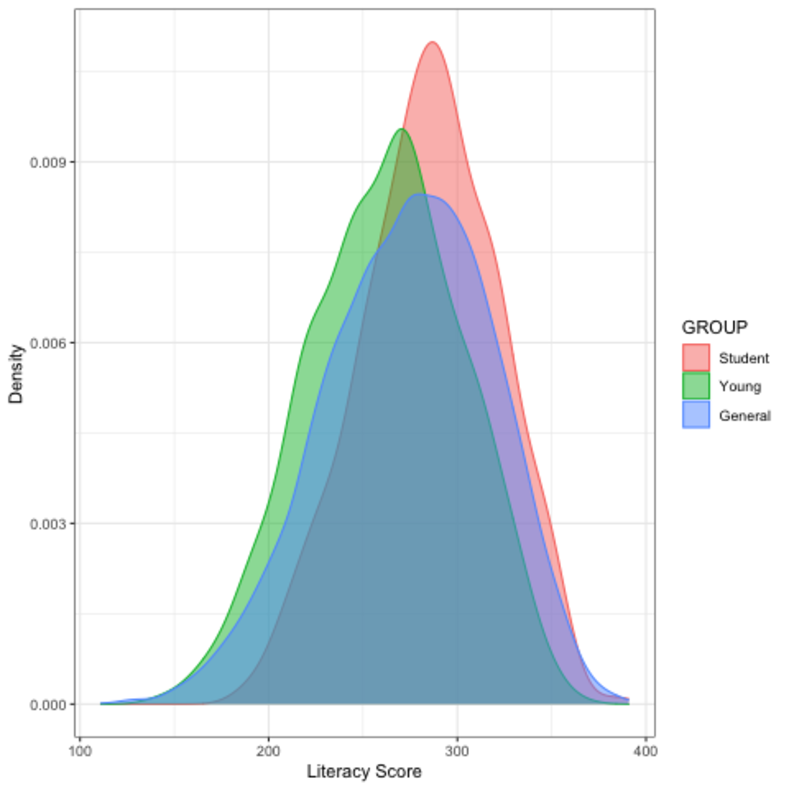

Supplement: S13 Fig — The red curve represents the Student sample, green represents the Young Sample, and blue represents the General sample. (TIF) [file pone.0271191.s013.tif]

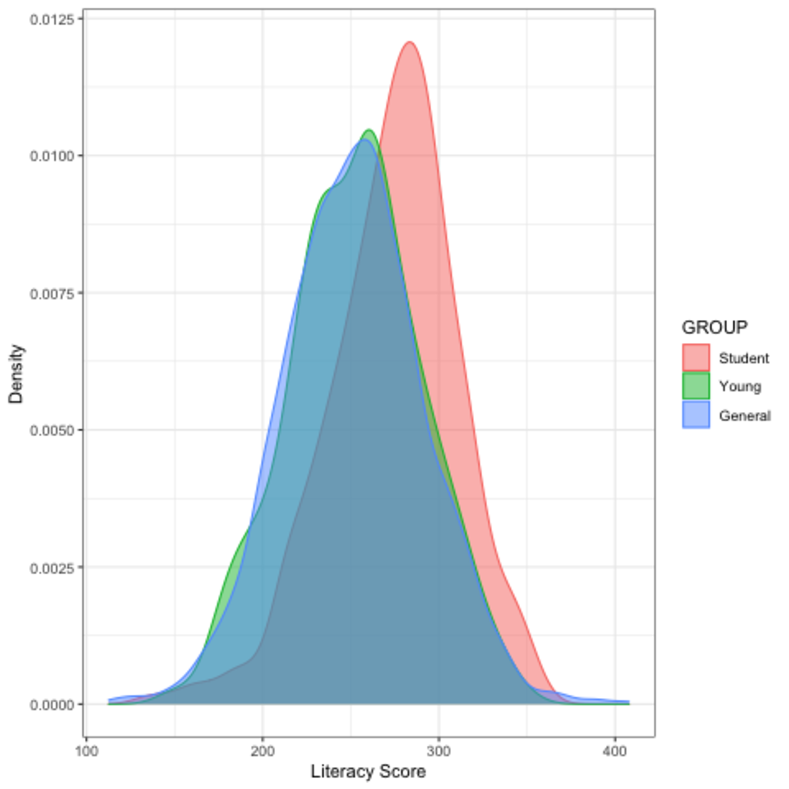

Supplement: S14 Fig — The red curve represents the Student sample, green represents the Young Sample, and blue represents the General sample. (TIF) [file pone.0271191.s014.tif]

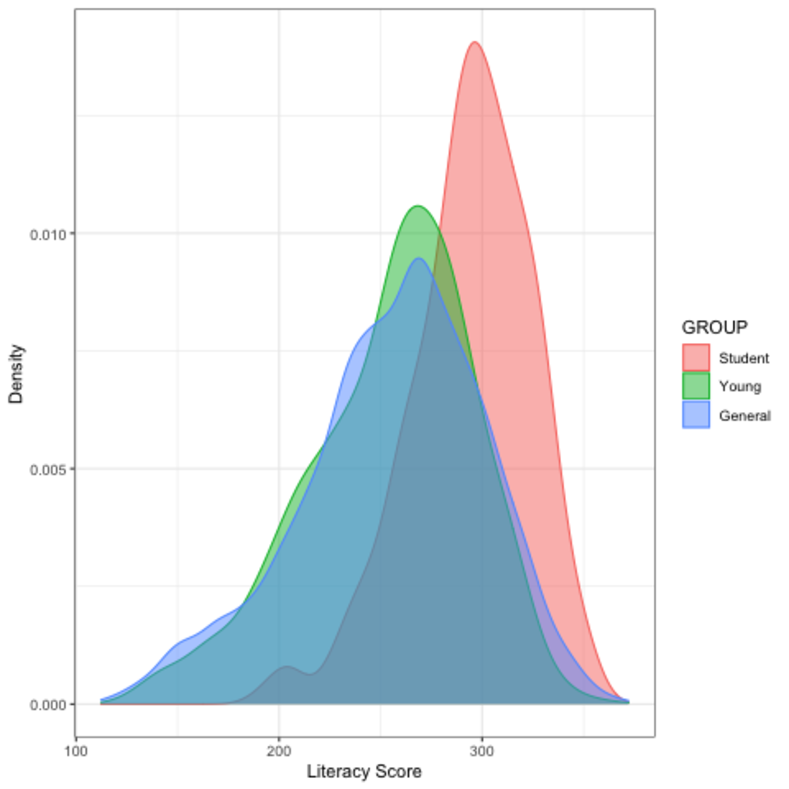

Supplement: S15 Fig — The red curve represents the Student sample, green represents the Young Sample, and blue represents the General sample. (TIF) [file pone.0271191.s015.tif]

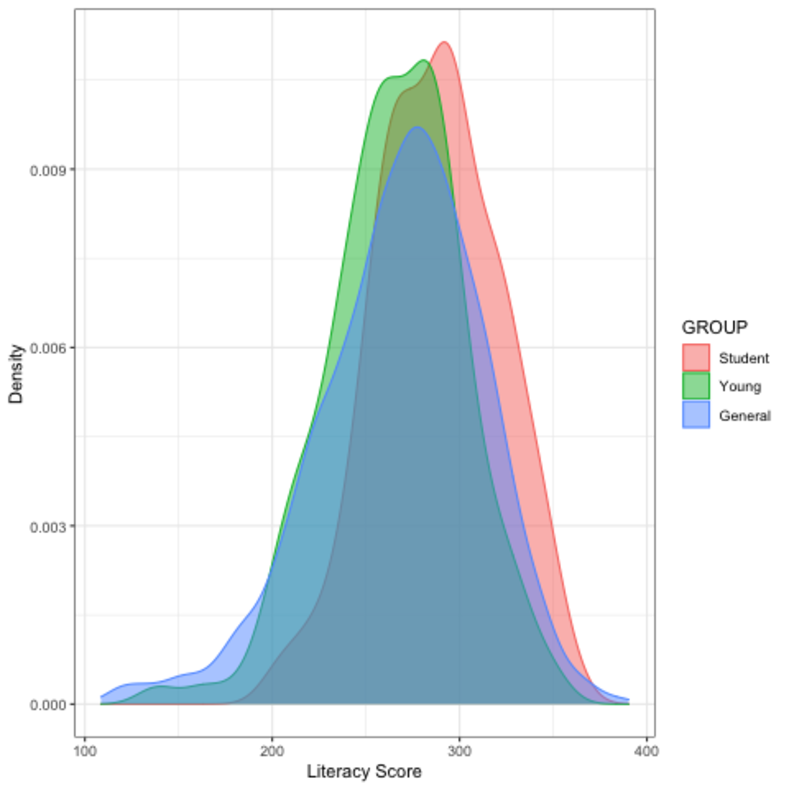

Supplement: S16 Fig — The red curve represents the Student sample, green represents the Young Sample, and blue represents the General sample. (TIF) [file pone.0271191.s016.tif]

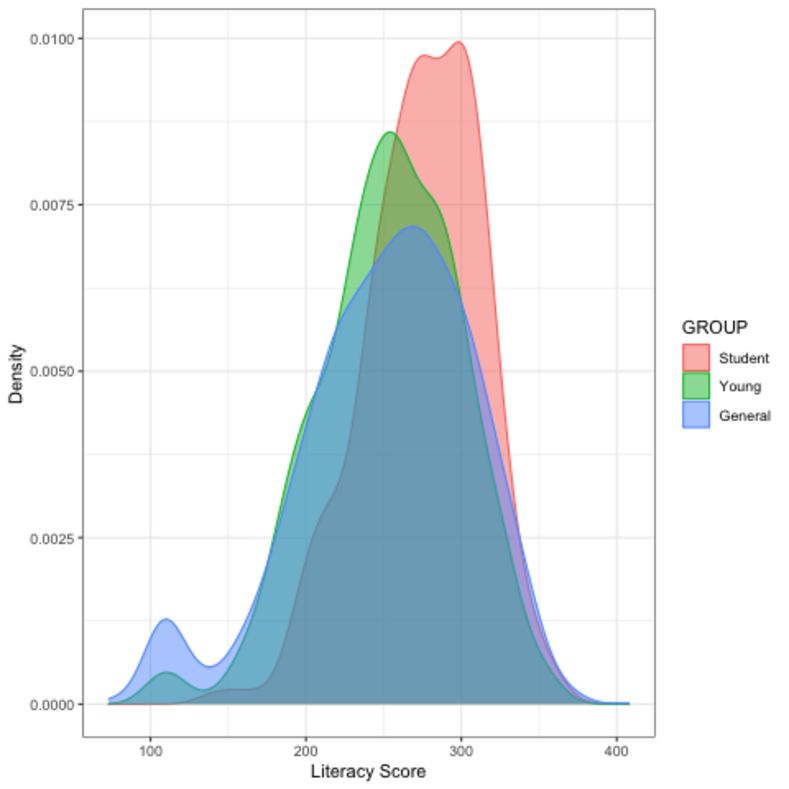

Supplement: S17 Fig — The red curve represents the Student sample, green represents the Young Sample, and blue represents the General sample. (TIF) [file pone.0271191.s017.tif]

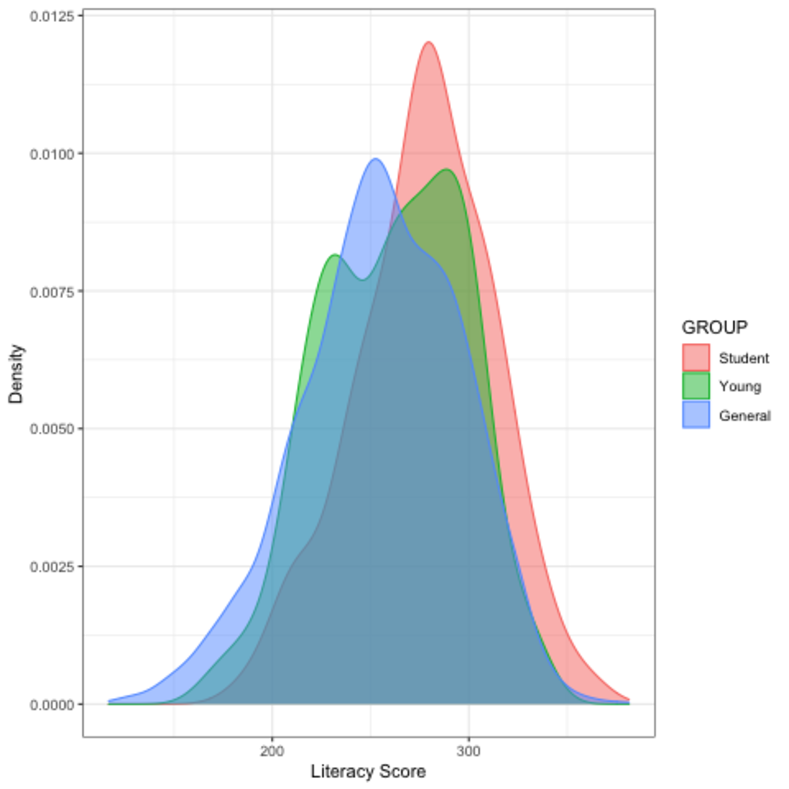

Supplement: S18 Fig — The red curve represents the Student sample, green represents the Young Sample, and blue represents the General sample. (TIF) [file pone.0271191.s018.tif]

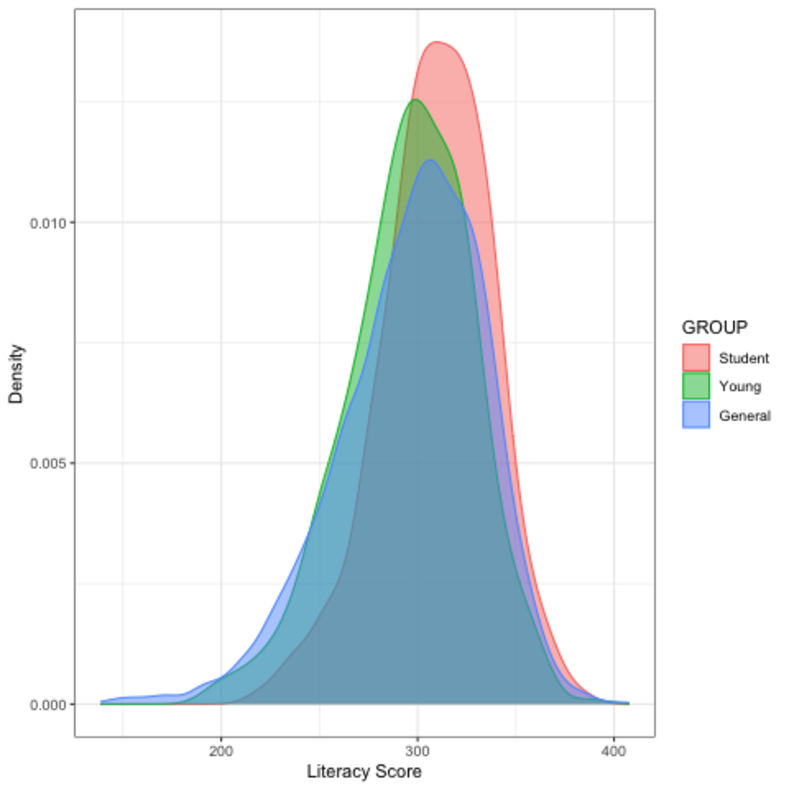

Supplement: S19 Fig — The red curve represents the Student sample, green represents the Young Sample, and blue represents the General sample. (TIF) [file pone.0271191.s019.tif]

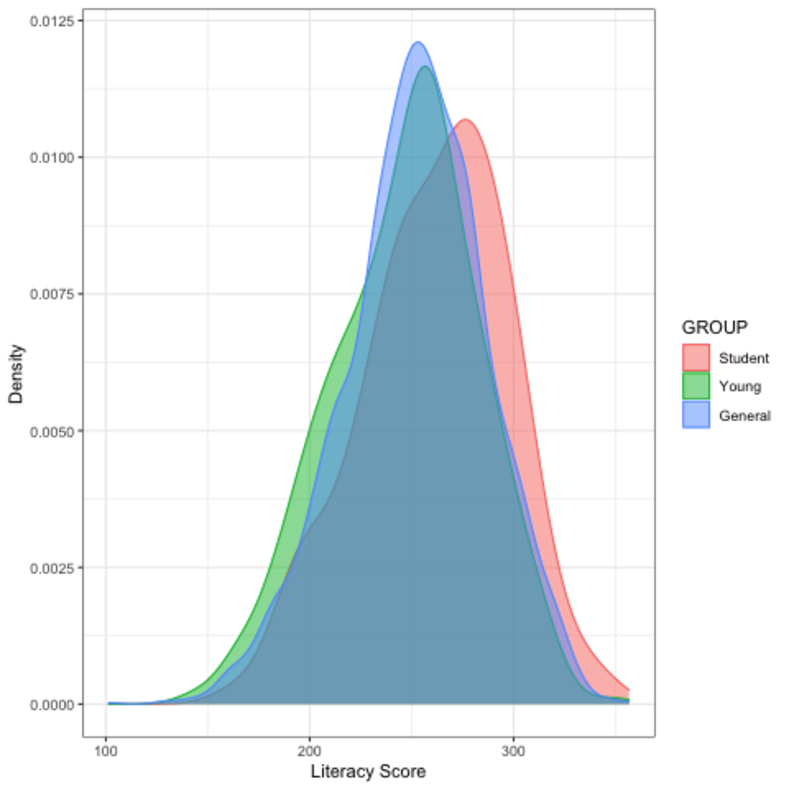

Supplement: S20 Fig — The red curve represents the Student sample, green represents the Young Sample, and blue represents the General sample. (TIF) [file pone.0271191.s020.tif]

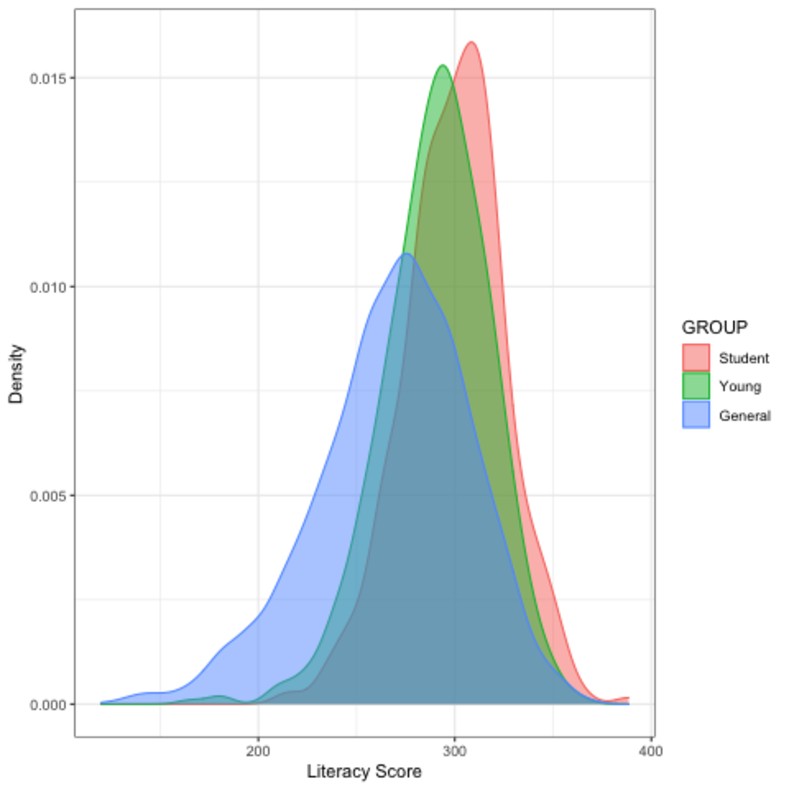

Supplement: S21 Fig — The red curve represents the Student sample, green represents the Young Sample, and blue represents the General sample. (TIF) [file pone.0271191.s021.tif]

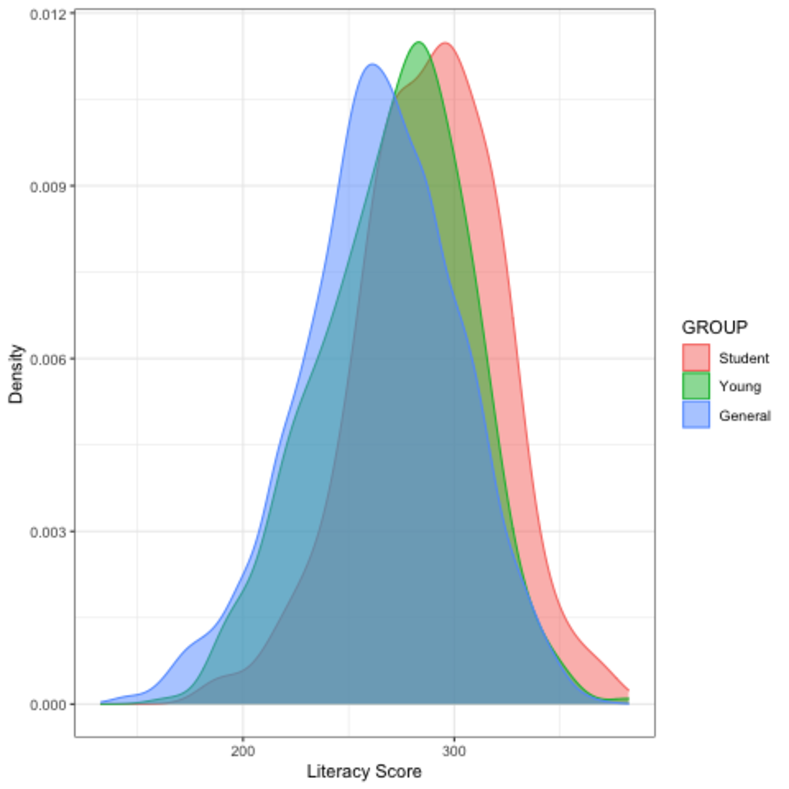

Supplement: S22 Fig — The red curve represents the Student sample, green represents the Young Sample, and blue represents the General sample. (TIF) [file pone.0271191.s022.tif]

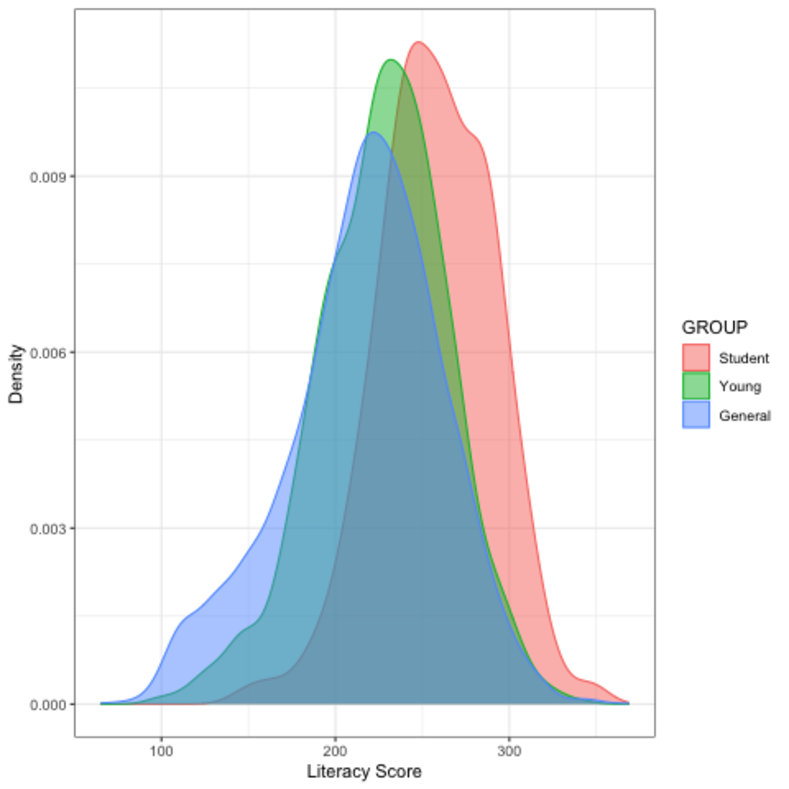

Supplement: S23 Fig — The red curve represents the Student sample, green represents the Young Sample, and blue represents the General sample. (TIF) [file pone.0271191.s023.tif]

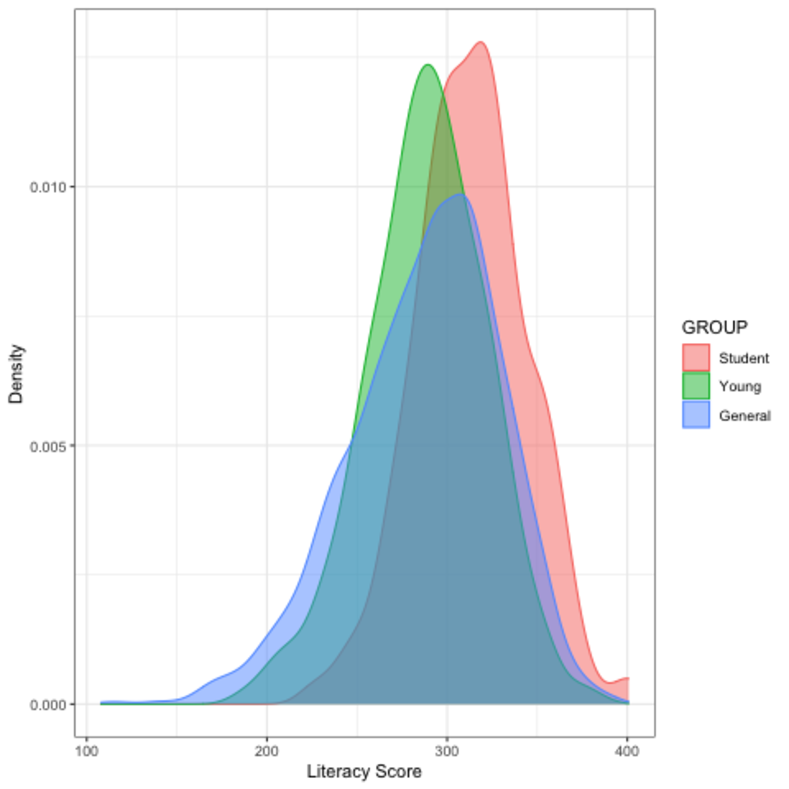

Supplement: S24 Fig — The red curve represents the Student sample, green represents the Young Sample, and blue represents the General sample. (TIF) [file pone.0271191.s024.tif]

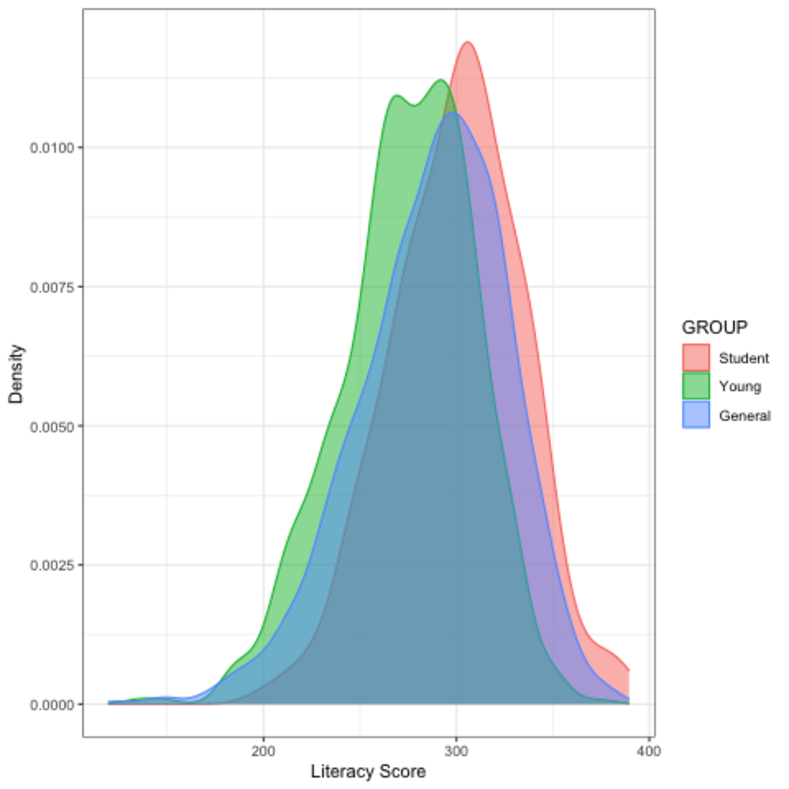

Supplement: S25 Fig — The red curve represents the Student sample, green represents the Young Sample, and blue represents the General sample. (TIF) [file pone.0271191.s025.tif]

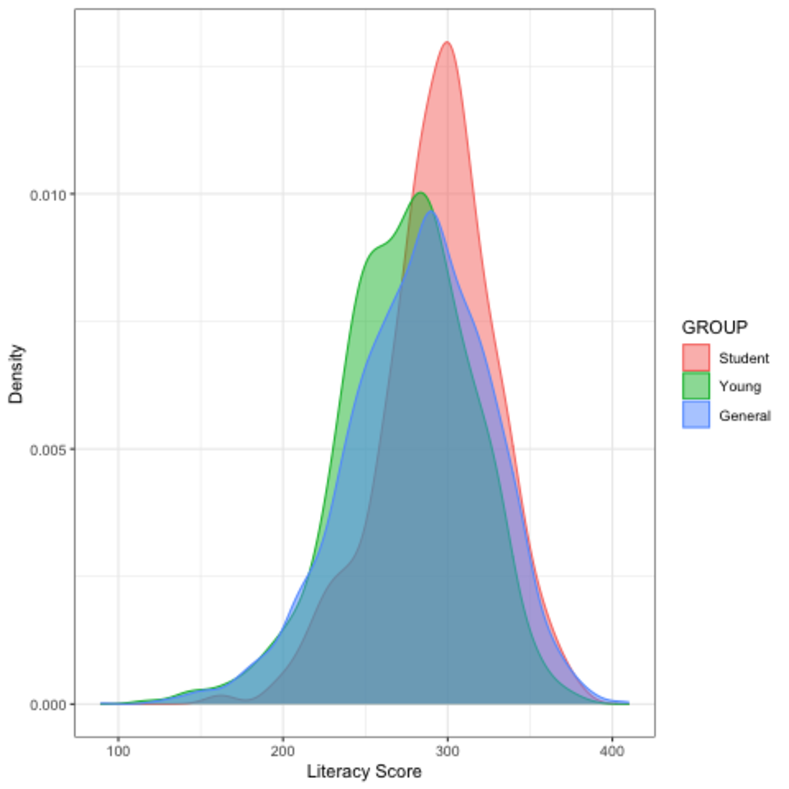

Supplement: S26 Fig — The red curve represents the Student sample, green represents the Young Sample, and blue represents the General sample. (TIF) [file pone.0271191.s026.tif]

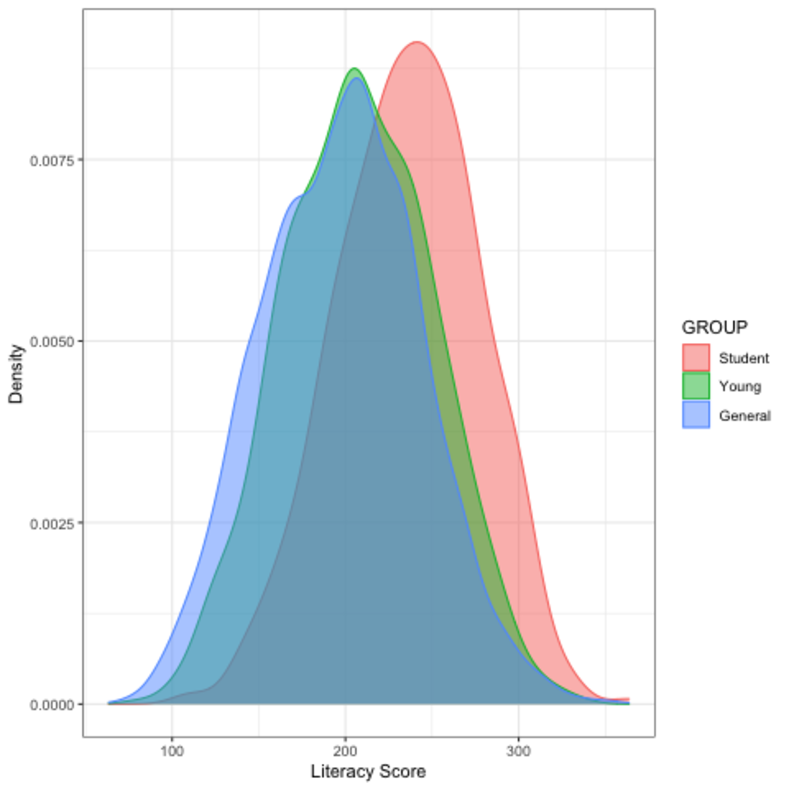

Supplement: S27 Fig — The red curve represents the Student sample, green represents the Young Sample, and blue represents the General sample. (TIF) [file pone.0271191.s027.tif]

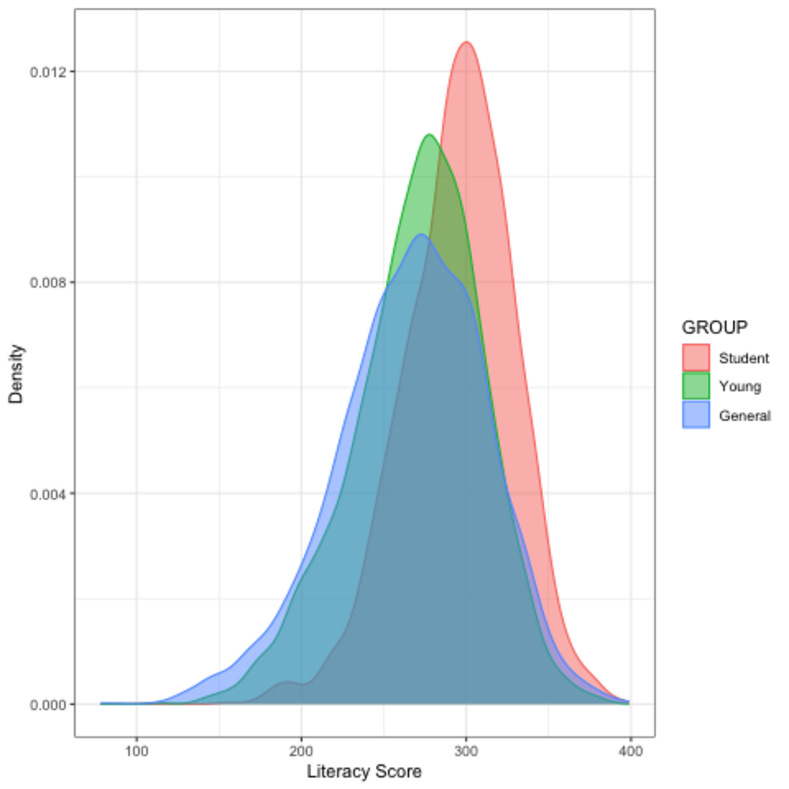

Supplement: S28 Fig — The red curve represents the Student sample, green represents the Young Sample, and blue represents the General sample. (TIF) [file pone.0271191.s028.tif]

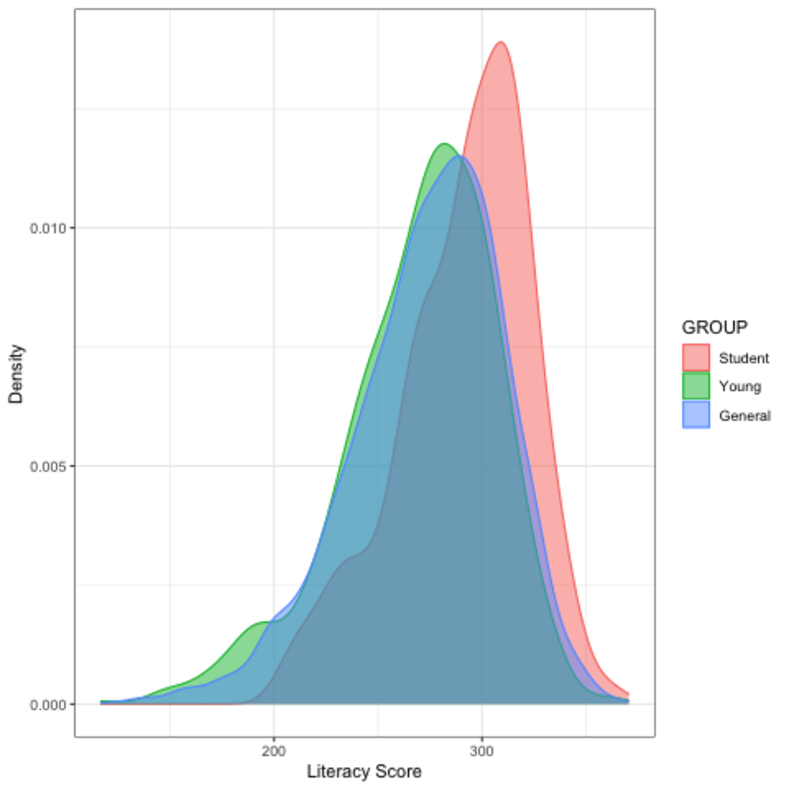

Supplement: S29 Fig — The red curve represents the Student sample, green represents the Young Sample, and blue represents the General sample. (TIF) [file pone.0271191.s029.tif]

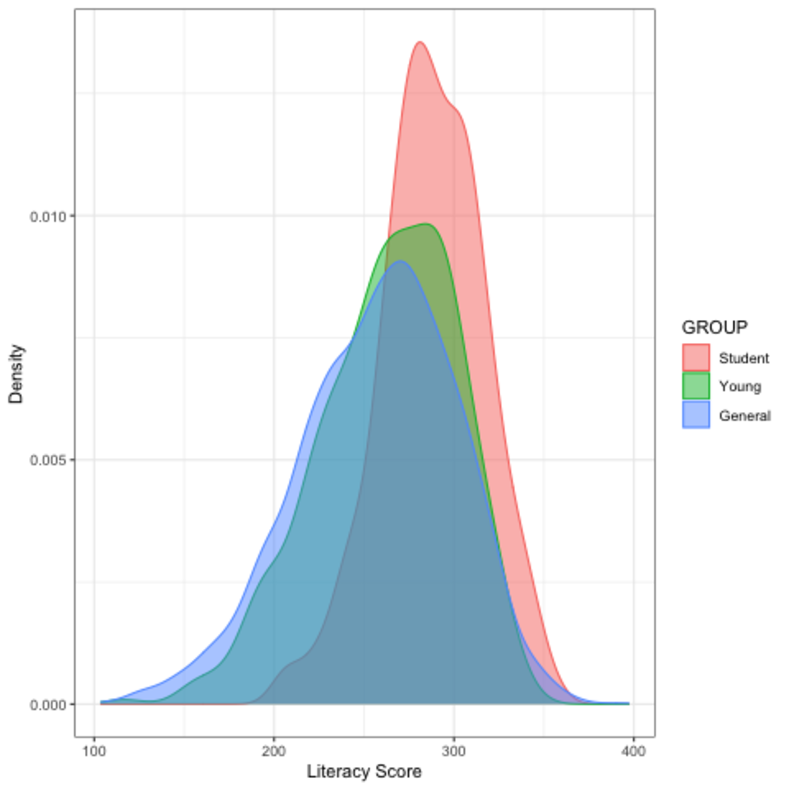

Supplement: S30 Fig — The red curve represents the Student sample, green represents the Young Sample, and blue represents the General sample. (TIF) [file pone.0271191.s030.tif]

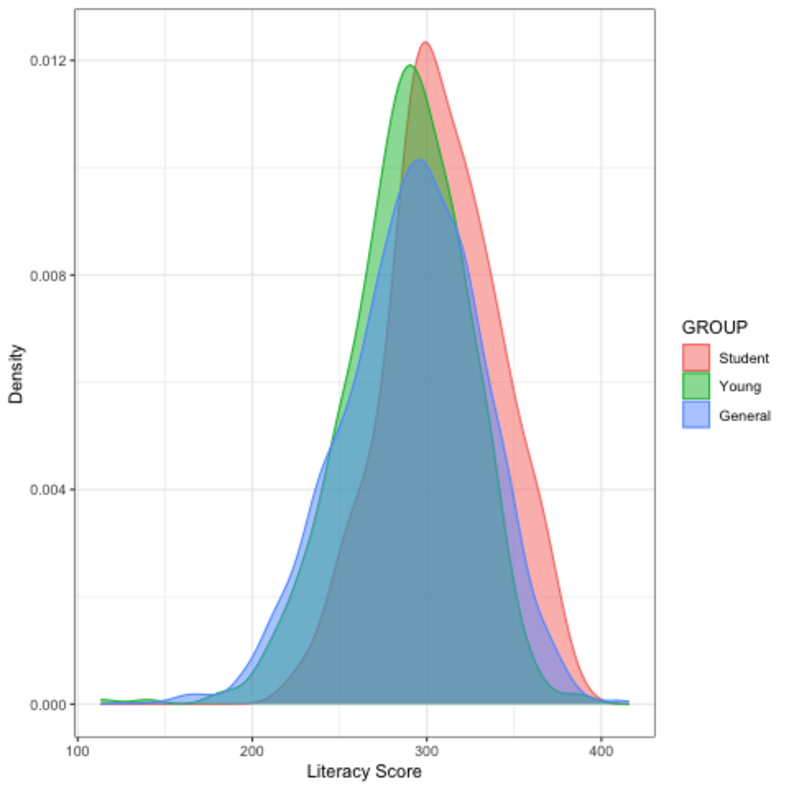

Supplement: S31 Fig — The red curve represents the Student sample, green represents the Young Sample, and blue represents the General sample. (TIF) [file pone.0271191.s031.tif]

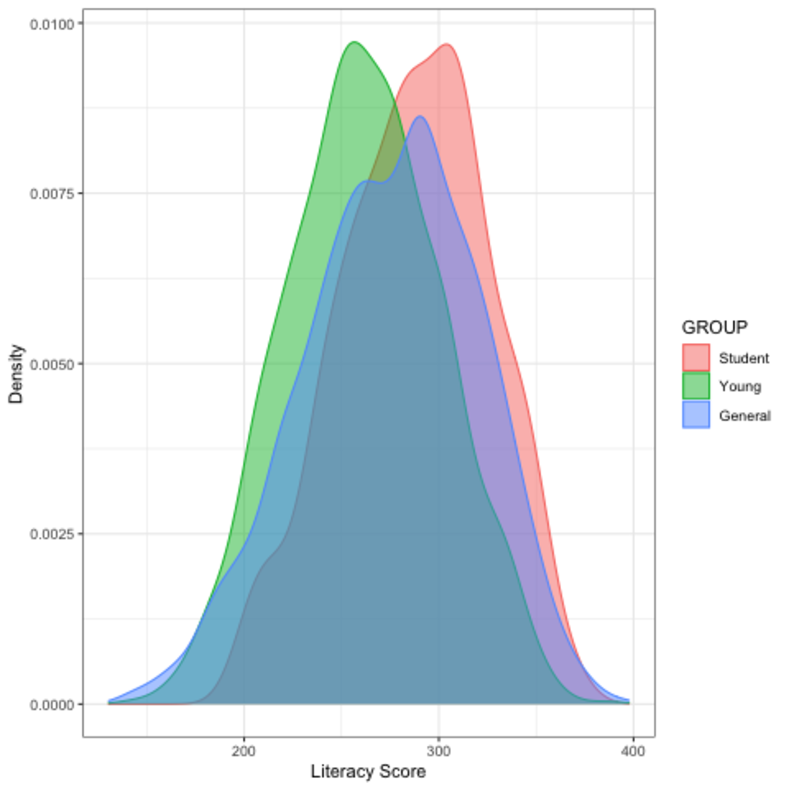

Supplement: S32 Fig — The red curve represents the Student sample, green represents the Young Sample, and blue represents the General sample. (TIF) [file pone.0271191.s032.tif]

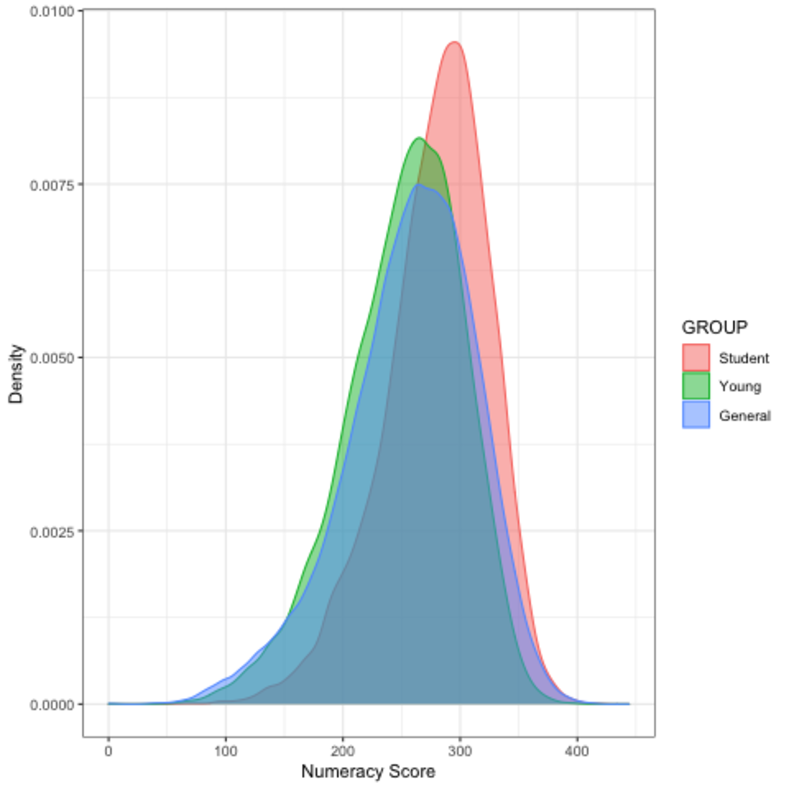

Supplement: S33 Fig — The red curve represents the Student sample, green represents the Young Sample, and blue represents the General sample. (TIF) [file pone.0271191.s033.tif]
